# The algorithm journey map: a tangible approach to implementing AI solutions in healthcare

**DOI:** 10.1038/s41746-024-01061-4

**Published:** 2024-04-09

**Authors:** William Boag, Alifia Hasan, Jee Young Kim, Mike Revoir, Marshall Nichols, William Ratliff, Michael Gao, Shira Zilberstein, Zainab Samad, Zahra Hoodbhoy, Mushyada Ali, Nida Saddaf Khan, Manesh Patel, Suresh Balu, Mark Sendak

**Affiliations:** 1 Duke Institute for Health Innovation, Durham, NC USA; 2 Harvard University, Cambridge, MA USA; 3 Aga Khan University, Karachi, Pakistan; 4 Duke University School of Medicine, Durham, NC USA

**Keywords:** Translational research, Sociology, Health care

## Abstract

When integrating AI tools in healthcare settings, complex interactions between technologies and primary users are not always fully understood or visible. This deficient and ambiguous understanding hampers attempts by healthcare organizations to adopt AI/ML, and it also creates new challenges for researchers to identify opportunities for simplifying adoption and developing best practices for the use of AI-based solutions. Our study fills this gap by documenting the process of designing, building, and maintaining an AI solution called SepsisWatch at Duke University Health System. We conducted 20 interviews with the team of engineers and scientists that led the multi-year effort to build the tool, integrate it into practice, and maintain the solution. This “Algorithm Journey Map” enumerates all social and technical activities throughout the AI solution’s procurement, development, integration, and full lifecycle management. In addition to mapping the “who?” and “what?” of the adoption of the AI tool, we also show several ‘lessons learned’ throughout the algorithm journey maps including modeling assumptions, stakeholder inclusion, and organizational structure. In doing so, we identify generalizable insights about how to recognize and navigate barriers to AI/ML adoption in healthcare settings. We expect that this effort will further the development of best practices for operationalizing and sustaining ethical principles—in algorithmic systems.

## Introduction

In the realm of healthcare and artificial intelligence, there is an abundance of conversations happening at the abstract level. We frequently discuss the potential^1^, policies^2^, and best practices of AI in a somewhat detached, hypothetical manner. While these discussions are important for shaping the future of healthcare, they often lack the crucial element of real-world grounding^3^. Meanwhile, the number of projects that reach the “clinical integration” stage has been growing; a survey of 95 randomized controlled trials (RCTs) of AI software found mixed results of their clinical impact^4^. Several works have provided high-level summaries about how to build ML models in healthcare, with particular emphasis on initial considerations for deployment^5^, ethical implications and considerations for regulation^6^, post-deployment evaluation criteria for projects^7^, legal and governance guidance^8^. As these case studies have developed, researchers have begun identifying “lessons learned” to help increase the adoption of AI software in health systems, including: how to conduct a silent trial^9^, reliability and fairness audits^10^, clinical workflow^11^, and change management and outcome monitoring^12^.

Moreover, when considering the adoption of new technologies in healthcare, historical examples remind us of the significance of moving beyond theoretical discussions. For instance, during the introduction of electronic health records (EHR), the benefits (e.g., flagging issues in medication orders) were carefully weighed against the hypothetical concern of alarm fatigue. However, it was only through the examination of specific case studies that unforeseen, complex scenarios came to light. A notable incident at UCSF serves as a stark reminder. Here, alert fatigue, coupled with a confusing user interface featuring different units for adult (mg) and pediatric (mg/kg) patients, resulted in a doctor accidentally ordering a 39x overdose, which was subsequently administered to a pediatric patient, nearly proving fatal^13^. Such a scenario is the result of a complex interaction of different decisions; no one could foresee that specific event, thus underscoring the value of studying real-world case studies in order to formulate sensible policies and best practices.

For that reason, it is valuable to create a workflow diagram to document the development of an ML-based tool for predicting sepsis in a hospital. Such a diagram serves as a tangible case study that can bridge the gap between theoretical discussions and practical applications of AI in healthcare. Workflow diagrams can empower stakeholders and function as communication tools for knowledge legitimation and diffusion. Sharing operational knowledge, or knowledge of day-to-day operations, allows stakeholders to navigate the organization and gain agency as they understand the outcomes and goals of their roles^14^. Furthermore, documenting on-the-ground processes creates a stable and tangible basis for knowledge building and legitimation^15^. Sharing and documenting knowledge about the full range of roles critical to technological work matches frameworks such as the Data Feminist^16^ principle to “Make Labor Visible.”

This study presents a comprehensive algorithm journey map (a set of workflow diagrams), capturing all social and technical activities involved in the procurement, development, integration, and lifecycle management of a health AI tool. Our contributions are as follows:

- We present the algorithm journey map of a Sepsis prediction tool at Duke called SepsisWatch and discuss our findings.
- We analyze these findings, particularly with an eye toward lessons learned in modeling assumptions, stakeholder recruitment, and organizational structure.
- We discuss limitations and future work.

Although this algorithm journey map is highly specific to the Duke SepsisWatch context, the exercise will be very valuable to other researchers both because it provides a blueprint for how one can build their own algorithm journey map and because even if a different organization doesn’t follow the exact same steps, there will be commonalities in the types of stakeholders, challenges, and enablers (e.g., institutional silos, differences in stakeholder priorities, technical barriers).

## Results

### How to read the algorithm journey map

The algorithm journey map is organized around four stages based on related work defining algorithm adoption stages^17^. The four lifecycle stages are:

1. *Problem identification*: How the organization identified sepsis as a problem that needed to be addressed and why a solution that uses AI is the best approach to address the problem. This stage ends with investing resources to build a sepsis AI tool.
2. *Development*: The building of the sepsis AI tool, preparing the clinical environment in which it operates, and designing the user interface and user experience. This stage ends with the decision to integrate the AI tool into clinical care. This stage zooms into two sub-stages, which are:
  - *Model build and validation*: building and validating a machine learning model on retrospective data
  - *User interface build and user experience design*: defining and developing the user interface and user experience.
3. *Integration*: Integrating the sepsis AI tool into the clinical environment and ends with a decision to continue using the sepsis AI tool after initial integration. This stage zooms into two sub-stages, which are:
  - *Technical integration*: integrating the technology into legacy systems and creating a way for the sepsis AI tool to run on real-time data
  - *Clinical integration*: integrating the sepsis AI tool into the clinical workflow
4. *Lifecycle management*: This stage describes post-rollout activities to manage, maintain, evaluate, and update the sepsis AI tool. This stage continues for as long as the AI tool is used in clinical care. It also includes monitoring the appropriate use of the tool and ensuring its decommissioning is initiated if it becomes obsolete or irrelevant.

**Fig. 1:**
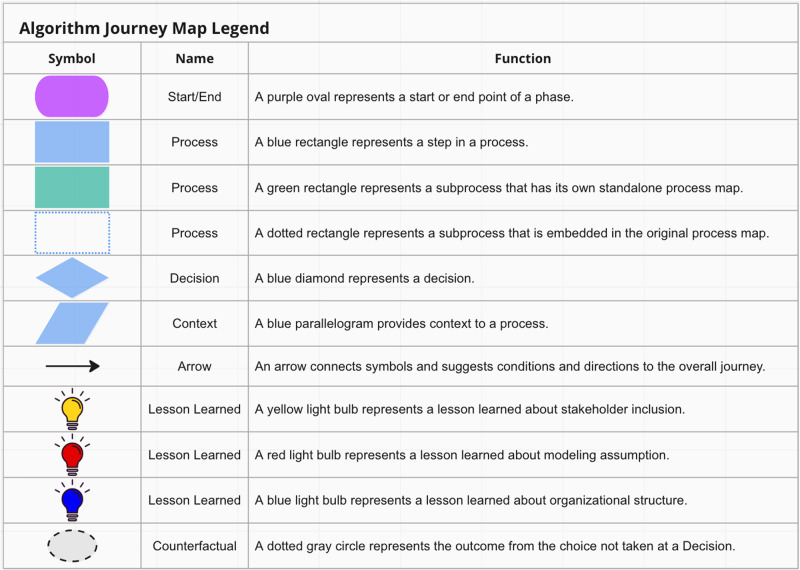
Symbols used in the algorithm journey map.

A full list of stakeholders mentioned throughout the algorithm journey map is identified in Table 1. We use the traditional event shapes from the swimlane literature—start/stop (oval), action (rectangle), and decision (diamond) using their canonical shapes from process maps^18^—and supplement them with some additional markers, all shown in Fig. 1. We introduce light bulb icons to denote ‘lessons learned’ that were identified by participants during interviews and dotted gray circles to denote “the path not taken” from each decision point. Due to the complexity of the multi-year effort, some processes are broken down into sub-processes; if a sub-process is complex and distinct enough we represent it with a green box and its own standalone map, whereas if it is small enough then we embed it in the original map but with a dotted blue line border.

**Table 1:** Description of each stakeholder listed in the swimlane diagrams

Role	Role type	Description
Clinical Champion	Clinical Lead	MD that proposed and co-led the project.
DIHI Project Manager	Technical Lead	Co-led the project. When the swimlane is just listed as “DIHI” the action is attributed to this role.
DIHI Statistician	Technical	Computer scientist that built and evaluated the model.
DIHI Data Engineer	Technical	Computer scientist that built a data pipeline.
DIHI UI/UX Designer	Technical	DIHI team members that designed prototype UI.
DIHI Clinical Expert	Clinical	MD that gave clinical expertise to data quality and use.
Health System Leadership	Leadership	Top decision makers (c-suite) for the health system.
University Leadership	Leadership	Top decision makers for the university. Does not have a healthcare-specific role.
Hospital Leadership	Leadership	Administrators for the hospital. Reports to health system leadership. Includes physicians and nurses.
Cardiology Leadership	Leadership	Administrators for the cardiology department, which previously housed the rapid response team. Reports to hospital leadership. Includes physicians and nurses.
Departmental Physician Leadership	Leadership	The Chair of the Surgery Department and Division Chief of the ED (which was a part of Surgery at the time).
Health System Nursing Leadership	Leadership	Chief Nursing Officer. One of the members of health system leadership.
ED RN Operations Leadership	Leadership	RNs who hold supervisory or management roles within an emergency department (ED).
Institutional Review Board (IRB)	Governance	Administrative body established to protect the rights and welfare of human research subjects.
Regulatory Affairs	Governance	Lawyers responsible for ensuring compliance with healthcare regulations.
RRT Nurse	User, Clinical	Specialized nurses who respond to medical emergencies within a hospital. Users of the Sepsis Watch tool.
Certified Nurse Educator (CNE)	Clinical	Nurses who have achieved certification in nursing education.
Health System IT Support	Technical	Technical employees who manage digital infrastructure to support care, data security, and administrative functions.

An explanation of how we created the algorithm journey map below is provided in the “Methods” section later in the paper.

### Algorithm journey map

**Fig. 2:**
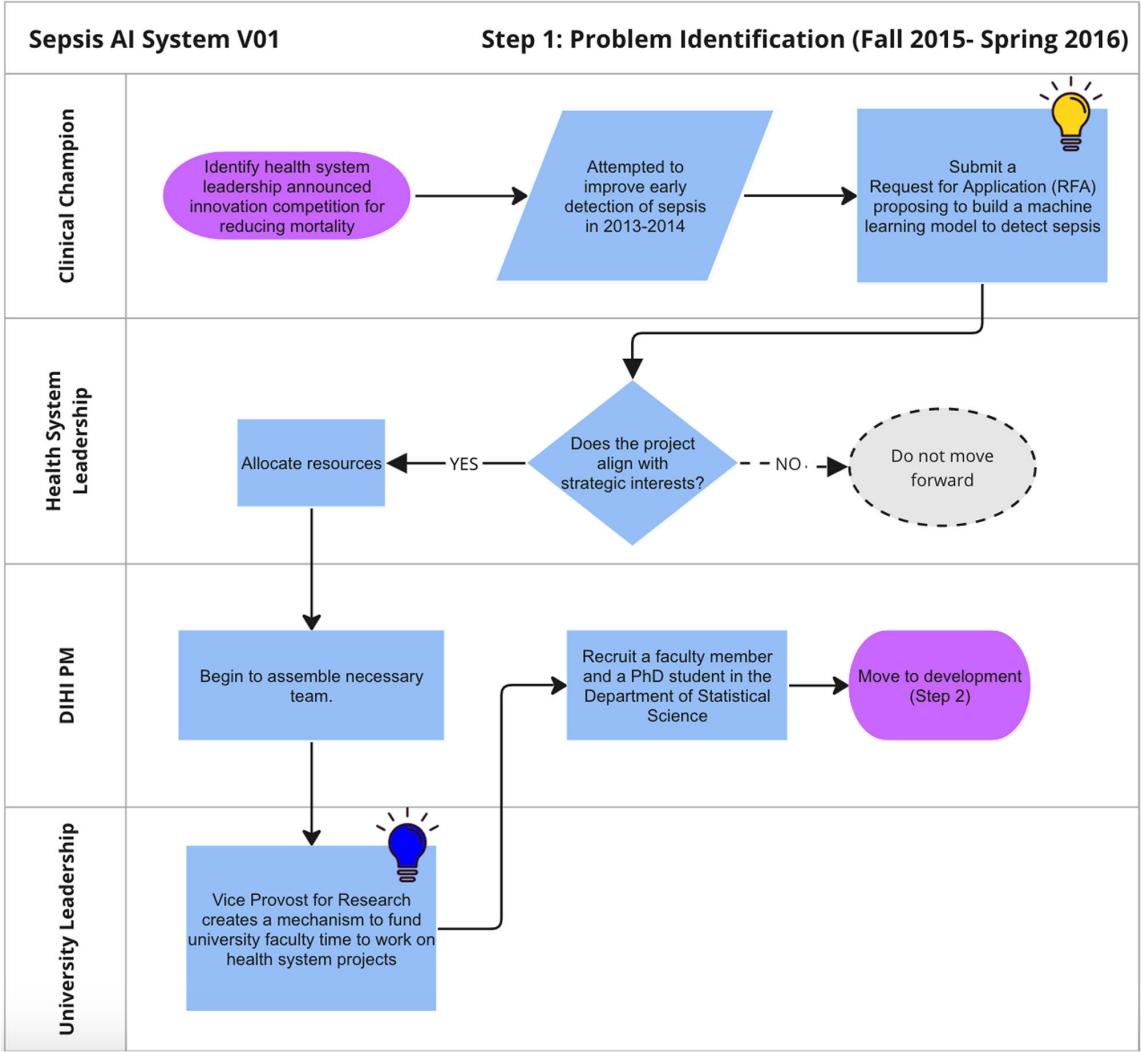
Journey map of problem identification phase.

Figure 2 shows the process of identifying and prioritizing the problem that led to the development and adoption of a sepsis AI tool. This process began in the fall of 2015 when health system leaders launched an innovation competition (i.e. the Request for Applications (RFA) process) that featured a strategic priority to reduce inpatient mortality. A small group of clinicians applied to the innovation competition and proposed to use machine learning to predict sepsis.

The proposal to develop a sepsis AI tool was selected by health system leaders for funding. Resources were allocated to pursue the opportunity, and staff from an internal innovation team were embedded in the project. In 2016, machine learning expertise within the school of medicine was limited and there was no mechanism for faculty in quantitative sciences departments to directly collaborate on operational health system projects. Health system leaders worked with the vice provost for research to establish a process whereby a statistics faculty and graduate student could dedicate effort to the sepsis AI tool project. The project team featured clinicians across specialties, project management, and statistics and machine learning expertise.

**Fig. 3:**
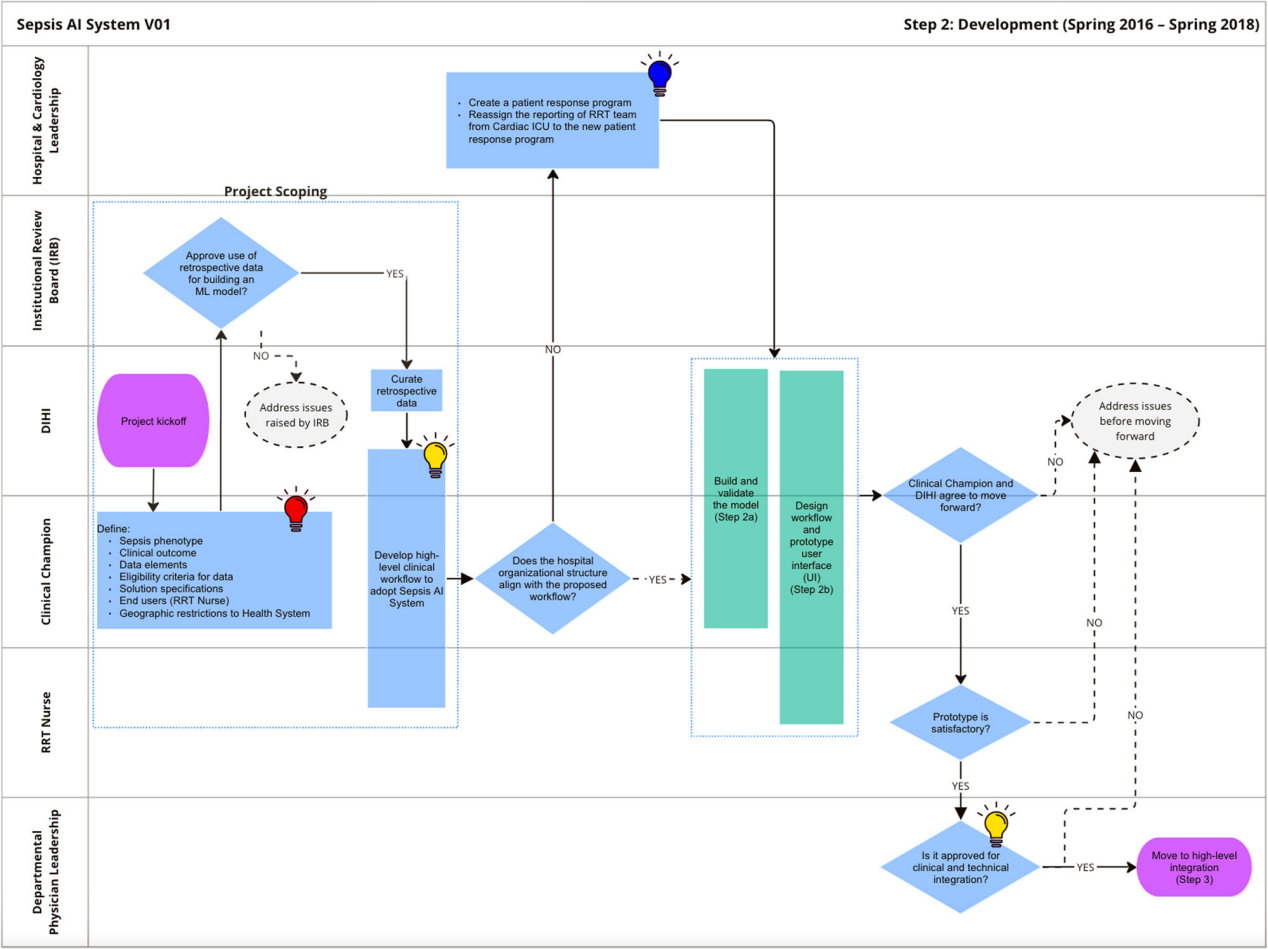
Journey map of the development phase.

Figure 3 shows the development stage. The innovation team project manager, in consultation with the clinical champion, guided the project through the many steps. During this stage, the clinical champions defined project goals and requirements, including:

- How is sepsis defined (e.g. CDC criteria, CMS criteria, presence of an ICD code)?
- What data elements are important for the predictive model?
- Who is the user (e.g. attending, resident, bedside nurse, rapid response team)?
- Which patients is the model run on (e.g. emergency department, all floors of main hospital, main hospital and regional partner hospital, ICU, non-ICU)?

The IRB reviewed the project to approve the development of the algorithm on retrospective data and granted a waiver of consent to use patient data for model development.

As will be described in the following sections, the above decisions guided the design and development of the sepsis AI tool and had significant downstream implications. Once it was decided that the rapid response team (RRT) nurse would be the primary user of the tool, the project team needed to ensure that organizational priorities would incentivize the tool to actually be used. RRT nurses were historically cardiac critical care nurses who supported care in the cardiac ICU when not responding to urgent events. These nurses reported to the cardiology service line, which was not primarily responsible for sepsis care quality. The project team worked with hospital leaders to create a new structure–the patient response program–that would house the RRT nurses and become responsible for sepsis care quality. During this restructuring, the clinical champion for the project became the patient response program director. These changes aligned RRT nurse management incentives with the objectives of the sepsis AI tool to improve sepsis care.

**Fig. 4:**
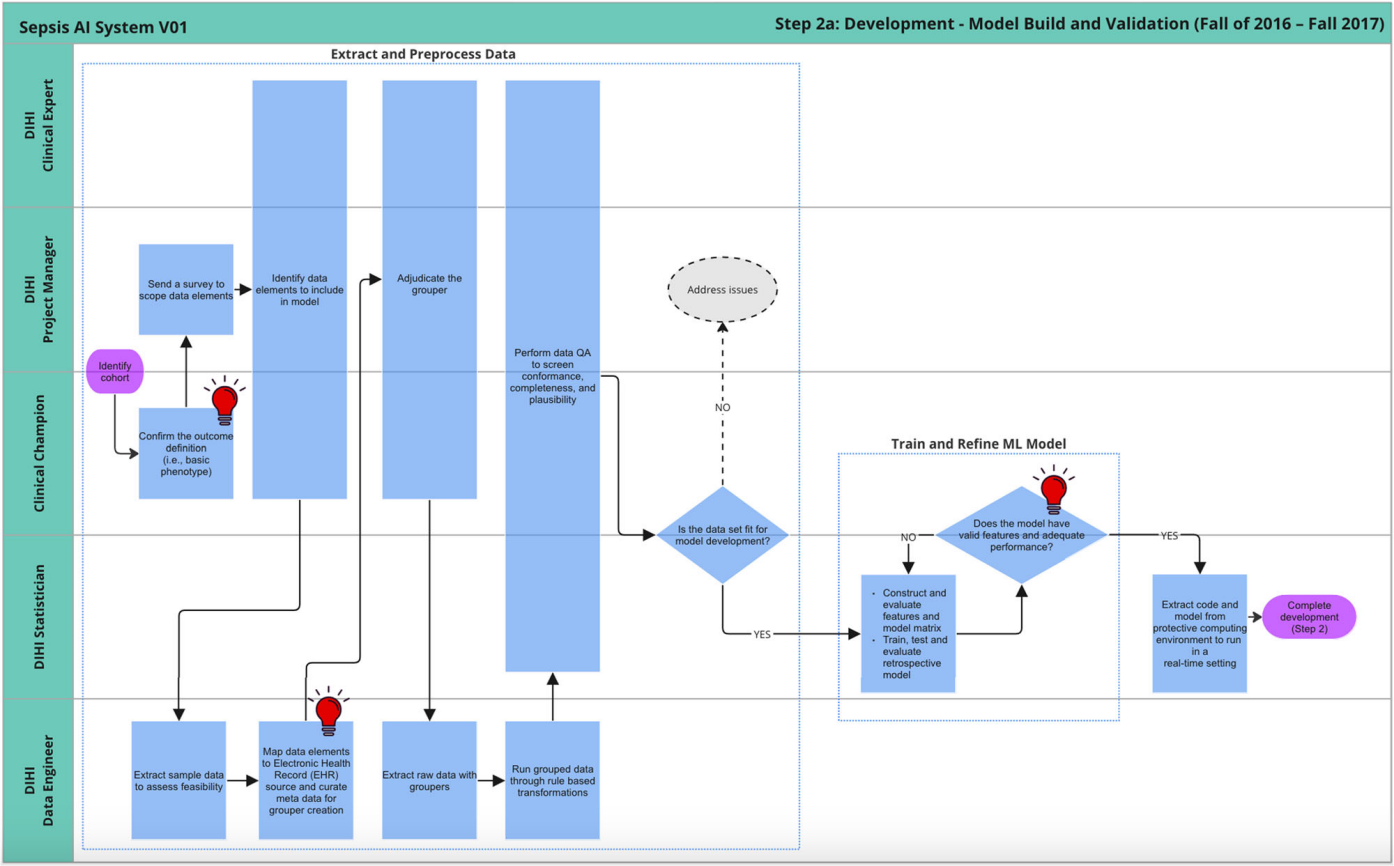
Journey map of model build and validation sub-phase of development.

**Fig. 5:**
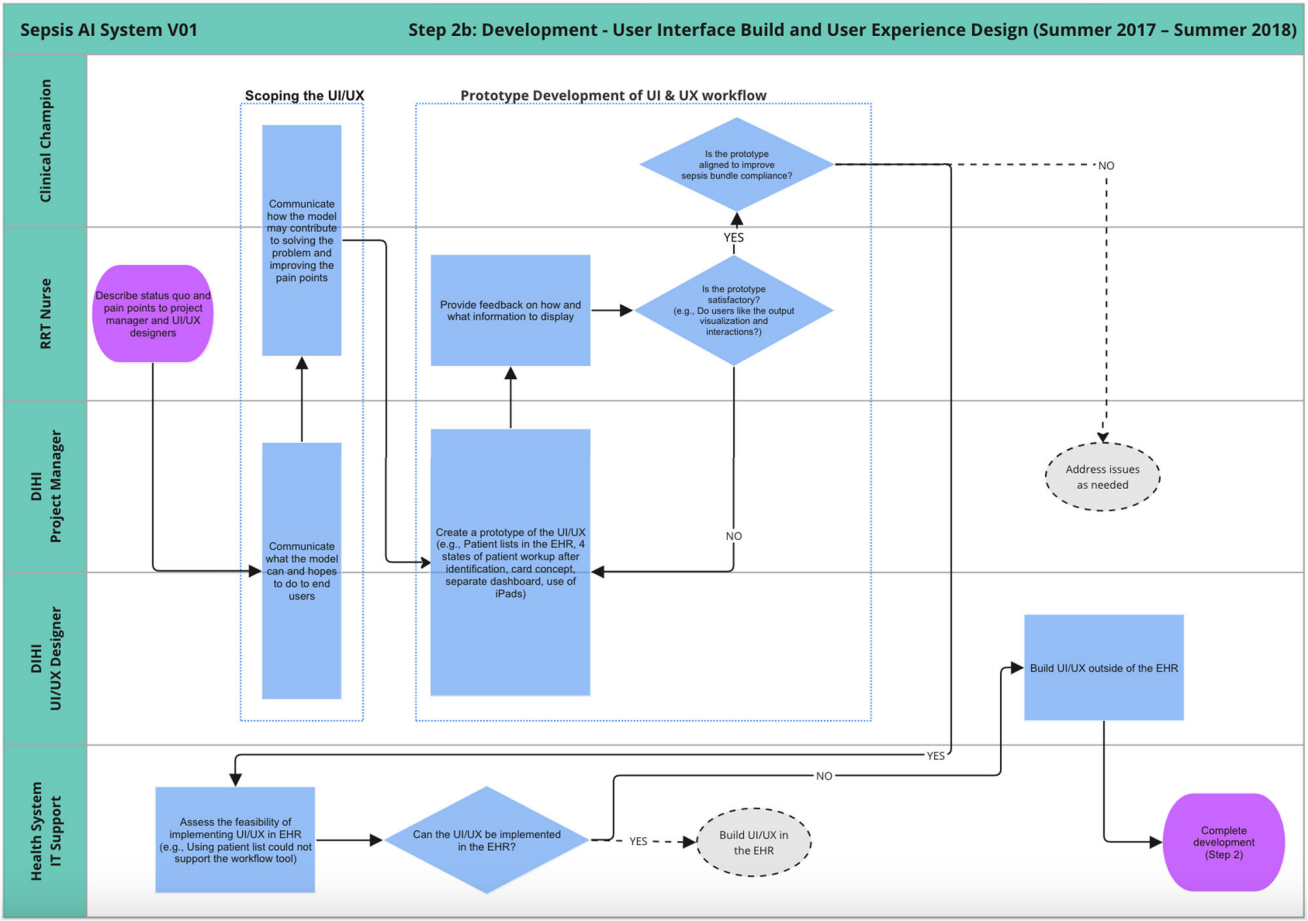
Journey map of the development of user interface and user experience.

Figures 4 and 5 detail the development of the sepsis predictive model and UI design, respectively. These processes are described in more detail below. Once these prototypes were built, department physician leadership reviewed the progress and approved moving forward with the integration process.

Figure 4 details the steps involved in building a machine-learning model on retrospective data. These steps are likely very familiar to machine learning model developers. After the team received cuts of historical data, the project manager worked with the clinical champions to clean the raw data. This included both grouping related raw elements (e.g., arterial blood pressure, blood pressure measured from left arm cuff) and performing quality checks to ensure the data aligns with clinical expectations. The data quality activities conducted for this project in 2016 - 2017 laid the foundation for a data quality assurance framework that was formally validated at a later date^19^.

Once the data engineer grouped and cleaned the data, the statisticians on the team built a machine-learning model, evaluating and refining it until it achieved sufficient performance on unseen data. The statistician then reviewed the output of the model with the clinical champion, both with summary statistics and chart reviews to assess whether the model was ready to move forward.

In parallel to the model building and validation sub-phase, Fig. 5 outlines the development of the UI design. This was an iterative process that began with scoping what the tool can help with based on the status quo workflow for delivery of care (i.e., reacting to sepsis once the patient already starts to deteriorate) and the general capabilities for what the tool can do (i.e., forecast who is at risk for deteriorating in the next N hours).

Next, there was an iterative design process where the UI designer would prototype ideas and discuss them with the end user (RRT nurses) for refinement. For instance, the original goal of the tool was just to flag high-risk patients in a dashboard, but the RRT nurses communicated that it would be even better if the tool helped them not just detect but also manage interventions to treat sepsis. That feedback resulted in reconceptualizing the AI tool as a “workflow tool” and not a dashboard. The UI designer and RRT nurse agreed on a workflow with four patient states (Triage, Screened, Monitoring, and Treatment) and the user would move the patients through the process as sepsis is detected and managed. This functionality could not be implemented in the hospital’s electronic health record at the time, so the team made the decision to develop an initial UI as a custom web application.

Another such example of iterative feedback involved model output visualization. Initially, a given patient’s predicted probability of sepsis was going to be plotted over time (to help remind the user of the patients they were keeping an eye on). However, after some feedback sessions with users, the UI designers began to worry that the users would use the trajectory/trend as an indicator, itself, and begin to over-rely on it. They concluded that such a scenario would require additional training for users to understand how to interpret time-based plots, so instead they focused on point-in-time visualizations to more closely match the setting the model was trained on without as large a risk of user misconceptions.

Once the iterative feedback was incorporated into the design, the prototype was presented to the clinical champion to ensure that the tool would be aligned with the project’s goals. In this case, the goal was both early identification of sepsis as well as timely treatment once identified. Treatment was to be measured based on sepsis bundle compliance as defined by SEP-1 sepsis bundle regulations issued by CMS^20^.

**Fig. 6:**
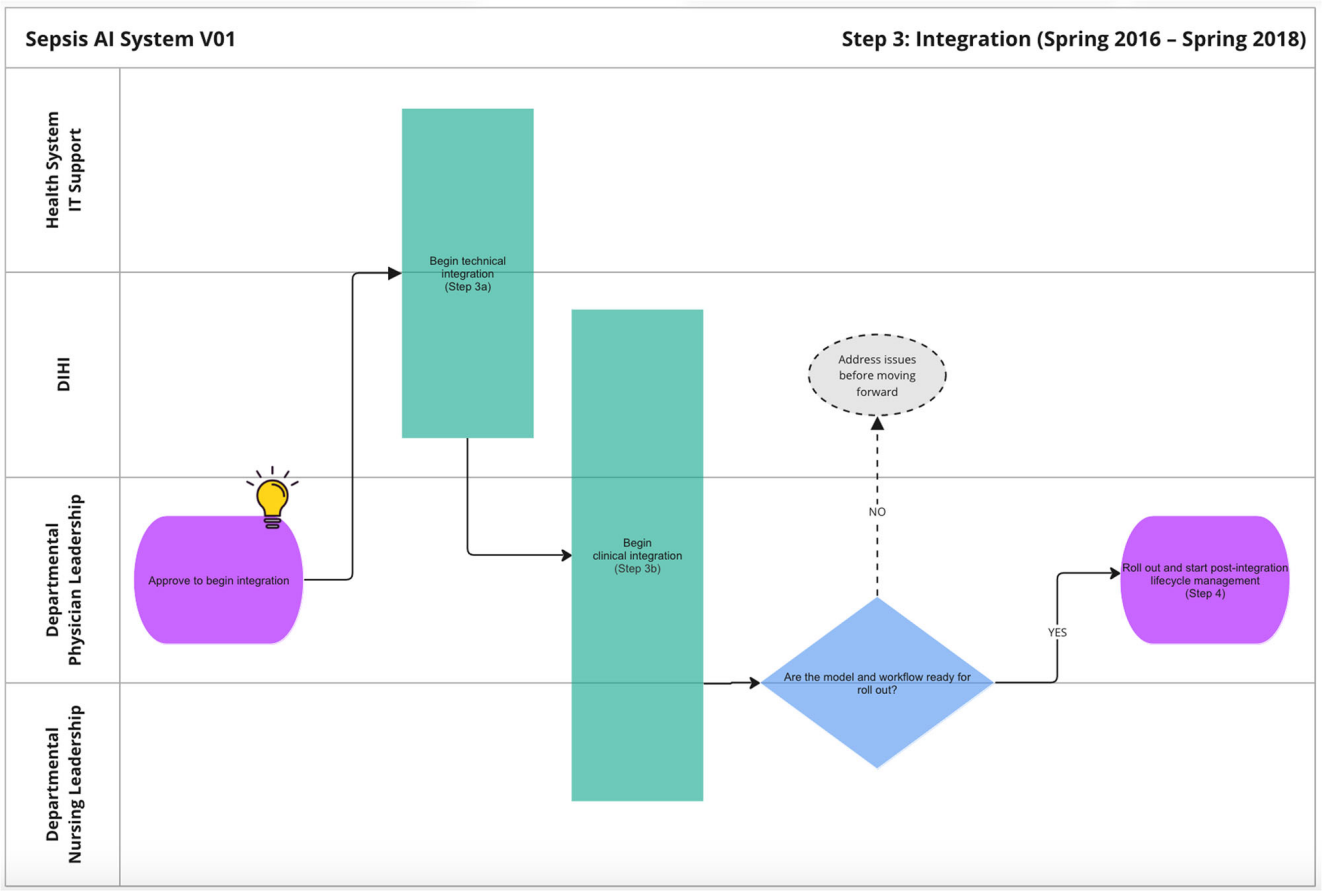
Journey map of the integration phase.

Figure 6 visualizes the next stage, integration. Integration contains two sub-stages, technical integration, and clinical integration, which are described in more detail below.

**Fig. 7:**
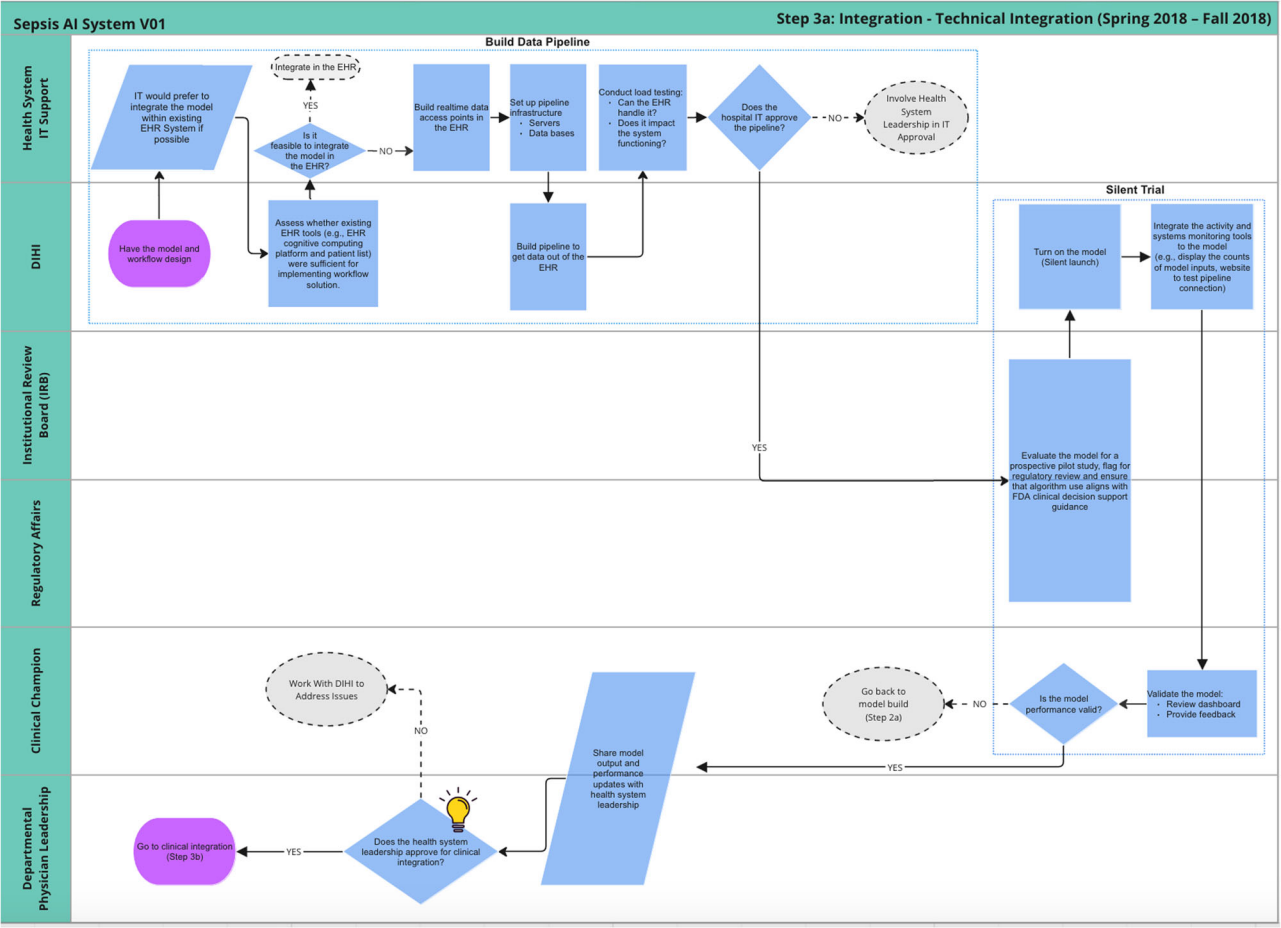
Journey map of technical integration sub-phase.

Figure 7 displays the process for technical Integration. It begins with extensive collaboration between the innovation team and the health system IT department. The teams navigated the tension between developing a fully customized solution, which would have higher maintenance and ownership costs and relying fully on existing tools, which would have lower maintenance and ownership costs. A major question that had to be addressed was whether the model could be integrated into Epic via its Cognitive Computing Platform (https://www.healthcareitnews.com/news/epic-cognitive-computing-platform-primer). Over a period of 6 months, the two teams conducted due diligence on the Epic solution and determined it was not able to run the sepsis AI tool. The teams agreed to develop a custom solution that extracts data from the EHR, pipes it to a server that runs the model, and sends those predictions to a database that displays results on a custom web application.

The IT and innovation teams built a data pipeline to extract data out of Epic’s Chronicles database in real-time. This required IT to build web endpoints to supply Epic data and the innovation team to build a schema for organizing the data that was received. Additionally, there needed to be resources for the server and database. A few months of testing were done to ensure the system could handle the volume of data being extracted, particularly because vitals are collected very frequently. Once IT signed off on the data pipeline, the sepsis AI tool was configured to pull real-time data once every 5 min. In addition, the innovation team built monitoring tools to regularly test the input/output connections and measure the volume of inputs.

Once the data pipeline was functioning, the innovation team submitted another study proposal to run a ‘silent trial’ to enable end-to-end system monitoring and testing. During this IRB review, the innovation team met repeatedly with regulatory affairs leadership to ensure that the sepsis AI tool aligned with the FDA’s definition of clinical decision support (CDS). Specifically, the relevant standards were based on the FDA’s “Clinical and Patient Decision Support Software” draft guidance which was posted in December 2017 (https://www.regulations.gov/document/FDA-2017-D-6569-0002). Because the tool did not make clinical decisions or treatment recommendations and supported independent review by clinicians, regulatory leaders determined that the technology qualified as non-device CDS. Once the ‘silent trial’ was approved, the innovation team conducted user testing to get feedback about the UI and the performance of the model. Once the user was satisfied with the changes, the innovation team presented the functioning tool to department physician leadership for approval. The clinical integration process began after approval.

**Fig. 8:**
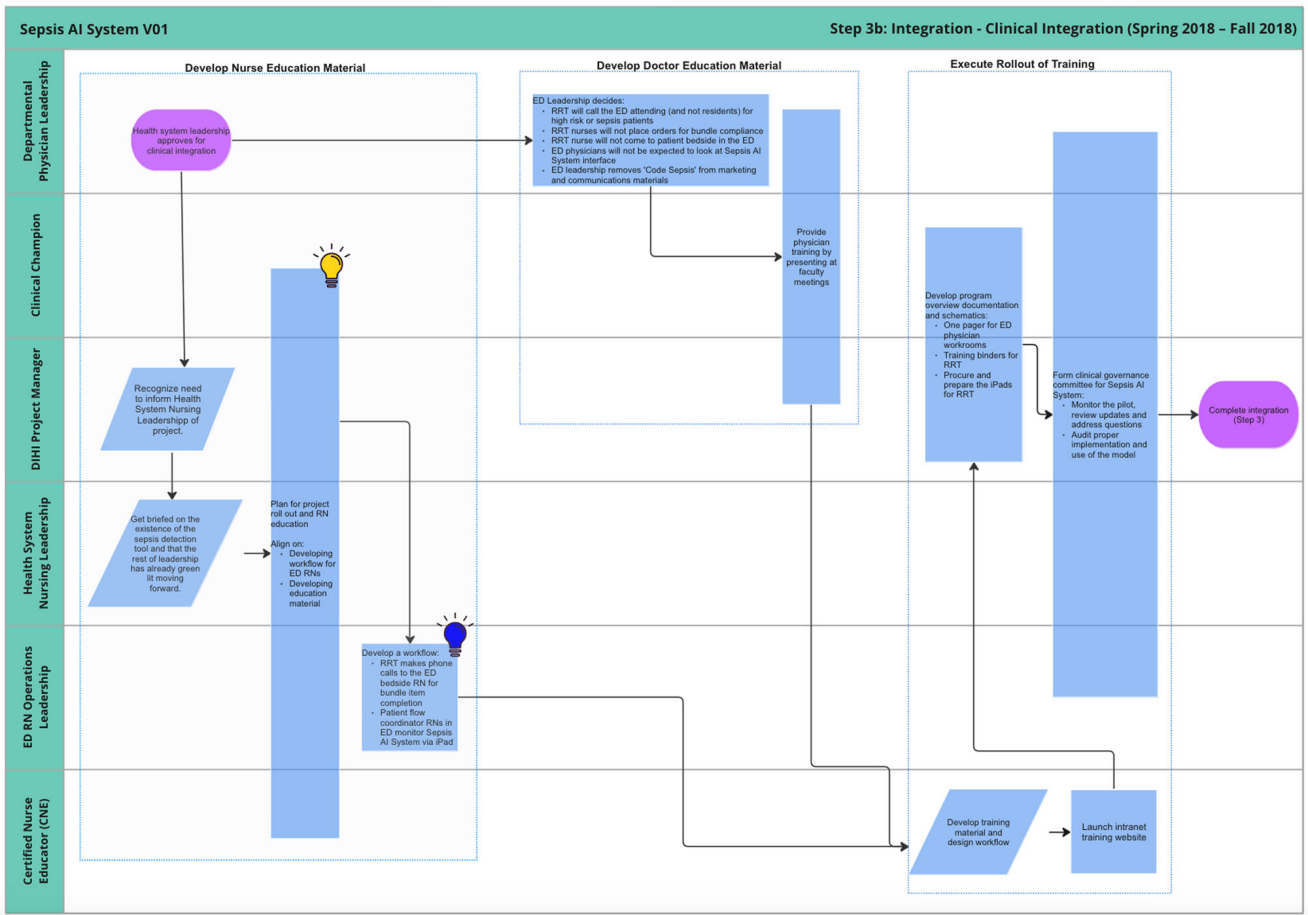
Journey map of clinical integration sub-phase.

Figure 8 visualizes the clinical integration process. This involved fine-tuning the workflow and user interface, developing training material, and assembling a governance committee. This began with two parallel processes, one for physicians and one for nurses.

The ED physician leadership finalized some decision points that hadn’t been fully specified, such as who the RRT nurse should call when the model predicts a high risk of sepsis (call the attending, not the resident), who will administer treatment (the bedside nurse, not the RRT nurse), etc. Additionally, there were some final suggestions for marketing and communications, such as removing any reference to a “code sepsis.”

At this stage, the innovation team met with the chief nursing officer for the health system to discuss the rollout of the sepsis AI tool. During this initial meeting, it became clear that there were additional stakeholders who needed to be engaged before the AI tool could be launched. Up until this point, the project team had been working primarily with physician leaders at both the hospital and department levels. Unfortunately, this left out nurse leaders at the health system level and within relevant service lines (e.g., emergency department) who needed to deploy resources to support the rollout. To address this lack of communication, the chief nursing officer convened a meeting with the innovation team, hospital nurse leaders, ED nurse leaders, and certified nurse educators (CNEs) to map out steps leading up to roll-out. Working closely with nurse stakeholders, several adaptations were made to the workflow, including direct communication between the RRT nurse and bedside nurses in the ED.

The innovation team worked with CNEs to develop training material, particularly for RRT nurses, to equip new users to appropriately use the sepsis AI tool. Finally, a governance committee was established including stakeholders from both physician and nursing leadership to meet monthly during the initial rollout in order to resolve any emergent issues.

**Fig. 9:**
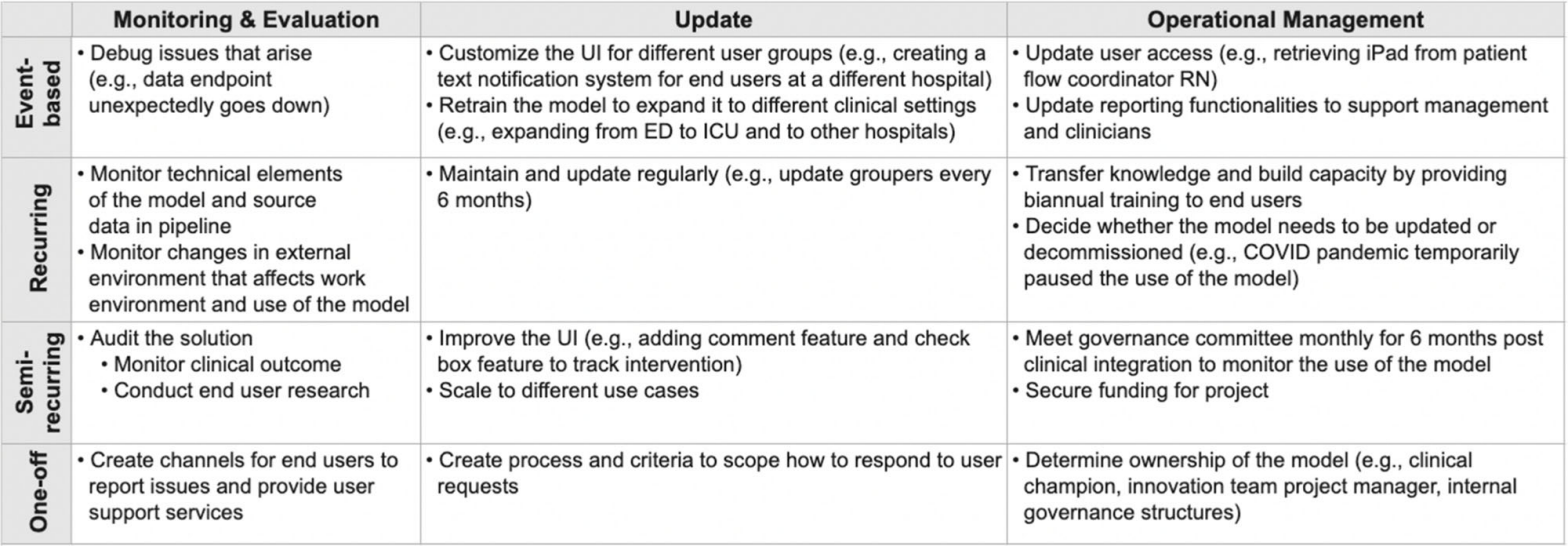
Different tasks that arise throughout post-rollout lifecycle management.

Unlike prior stages, post-rollout lifecycle management is not a linear-flow process. Some tasks are predictable whereas others are responsive to events that occur (e.g., user requests, technical failures, etc). Instead of employing a swimlane-oriented diagram to convey lifecycle management, Fig. 9 shows a variety of different activities, categorized by both type of task (monitoring, updates, and operational management) and frequency (one-off, semi-recurring, recurring, and event-based). These activities also involve many stakeholders, principally driven by the project manager and clinical champion.

Monitoring involves both regular dev-ops duties (e.g., is the system still up? Were there large changes in data volume?) as well as periodic, thorough data science analysis (e.g., does the model still perform well? Have clinical outcomes improved?). Monitoring and evaluation are a technical component of system audits, assessing model performance as clinical outcomes, and process measures over time. These audits have also involved user research, such as shadowing the RRT nurses who use the tool. Additionally, CMS requires hospitals to conduct external auditing of bundle compliance in order to maintain eligibility for Medicare payments.

Updates are performed both as needed (event-based, semi-regularly) and on a regular schedule (recurring) in order to ensure the robust performance of the sepsis AI tool. An example of a recurring update that occurs every 6 months is a coordinated effort between the innovation team and clinical champions to refresh data element “groupers.” This process ensures that any new medications, vital sign monitors, or laboratory equipment are accounted for in the data pipeline. An example of a one-off update responding to a user request was adding new functionality to automatically identify treatment bundle compliance in real-time. Although there is no formal process, when users request new features, the project team must categorize the feature as either an ‘update to the existing product’ or a ‘new project that needs separate, dedicated effort.’ An example of a task that spun off into a standalone product is the alert notification system that now supports many AI tools.

Operational management involves ongoing ownership and accountability. The sepsis AI tool has 2 “owners”—the clinical champion and the innovation team project manager—who communicate with each other and liaise with additional members to support the sepsis AI tool as needed. They periodically need to secure funding and resources for the project and assess how well the solution is addressing the original objective. The project owners also ensure that existing and new staff are appropriately trained and that training material is maintained to reflect evolving standards of care for sepsis. Additional entities also play an active role, such as how institutional leaders are now seeking input from the FDA to ensure that the use of the tool continues to comply with the intention of non-device CDS in light of the 2022 final CDS guidance (https: //www.fda.gov/regulatory-information/search-fda-guidance-documents).

### Interviewee-identified opportunities for improvement

Throughout the construction of the algorithm journey map, we asked interviewees to identify not just what happened but also what they might have done differently in retrospect. This includes both narrow/technical and broader opportunities, and such reflections were indicated in the algorithm journey maps with lightbulb icons. The icons were separated into three categories: modeling assumptions (red), stakeholder inclusion (yellow), and organizational structure (blue). Below, we highlight specific learnings from the journey map and also abstract generalizable insights that can inform other efforts to develop and integrate AI into clinical care.

#### Modeling assumptions

There were multiple areas where technical decisions about the model hampered the project. Most of these decisions occurred early in the algorithm journey:

- *[Development] Scoping the solution*: Early on, clinical collaborators at that time felt the sepsis AI tool would not be used in the ICU, so data was thus truncated at the time of ICU transfer. This single decision limited the future ability to expand the use of the sepsis AI tool beyond the ED to general inpatient wards. When a user requested to expand the use of the AI tool to inpatient wards, a new “2.0” project had to be initiated. Generalizable insight: Carefully consider the downstream impact of inclusion and exclusion criteria applied at the level of patients and individual data values. If the use of an AI tool may extend to adjacent use cases, make sure that relevant data is included in model training and evaluation.
- *[Model Building] Outcome definition*: The project team did not initially appreciate the difficulty in finding the “right” definition of sepsis. Physicians had differing opinions about which outcome to use, and the published literature didn’t show consensus. Modeling became easier once the outcome definition was modularized, allowing for easily changing the criteria and retraining the model. Generalizable insight: Do not limit outcome labels to single sets of criteria and develop (and validate) models for multiple types of definitions. Even if there is a professional consensus today on how a disease is defined, anticipate future changes.
- *[Model Building] Real-time access to model inputs*: When determining which data elements to include as inputs for the model, the team had not initially considered that any data for the model needed to not just be captured in the EHR but also available in real-time. Epic’s backend databases involve both a real-time feed of the current day and a historical archive, and access to real-time data requires the IT department to build specific data endpoints for each kind of element. Generalizable insight: Only include data elements in an AI tool if the data is available and robustly captured at the time predictions need to be made.
- *[Model Building] Environment constraints on model*: Initial versions of the model involved a technically complex Multi-task Gaussian Process for data imputation, followed by an LSTM classifier. The plan had been to integrate this model into production, but during technical integration, the team realized this approach required matrix inversion and significant computation. Eventually, the team used fill-forward data imputation for the LSTM, but it should have been knowable at the time that the runtime environment would limit expensive model architectures and decisions. Generalizable insight: Plan for ablation studies that evaluate the impact of removing model components or input features. When building an AI tool for integration, reduce unnecessary complexity.

#### Stakeholder inclusion

Beyond technical lessons, there was another ‘obvious’ insight from the mapping exercise. Many decision points throughout the process (e.g., problem formulation, workflow design, signing off with integration) were shaped and approved by hospital and department leaders who were physicians, but not nurses. This culminated in the clinical integration stage being nontrivially complex and stressful. This oversight also created tension between different clinical stakeholders that needed to be addressed leading up to a large project launch.

Although the RRT nurse users were included in the early designs, it was not well understood that physicians and nursing leaders within service lines and hospitals manage separate activities. The innovation team needed to be directly engaging leaders across both chains of command, rather than expect communication between the two groups. As a result of this oversight, the clinical integration stage involved meeting many levels of nursing leadership (health system-level, hospital-level, and department-level) as well as directly engaging certified nurse educators to finalize programmatic decisions and develop training material.

The yellow light bulb icons indicate all of the opportunities where nurse leadership could be (or eventually was) involved in the project approval.

The generalizable insight from this lesson is to identify up-front the reporting structures, training requirements, and communication channels for all clinical professions affected by an AI tool put into practice. Even if clinicians across professions appear to work closely together in the same unit, reporting, training, and communication channels may be distinct. Project leaders also cannot assume that information shared with front-line workers or business-unit leaders is shared upwards within reporting structures. Executive leaders need to be informed and have their concerns addressed prior to the integration of new AI tools.

#### Organizational structure

One final set of learning opportunities comes from identifying commonalities around structures and workflows. These events were not about what should have been done differently in the moment but instead flagged organizational changes that took a great deal of effort and could be streamlined. The following learnings are highlighted with blue lightbulb icons:

- *[Problem Identification] Recruit statisticians*: The innovation team partnered with a faculty and Ph.D. student from the statistics department of our organization’s university. However, there was no mechanism for research faculty to dedicate time to health system projects. The Vice Provost for Research at the university helped facilitate the collaboration. Since that time, the innovation team has grown significant internal machine learning expertise in order to move more quickly on projects. Generalizable insight: Ensure senior-level support to engage perceived outsiders in the development of AI tools used in clinical care. Even if technical expertise exists within the organization, trust must be established between senior technical and clinical leaders.
- *[Development] Create a patient response program*: As discussed in greater depth earlier, the project team needed to ensure that organizational incentives enabled RRT nurses to prioritize the use of the tool. This involved working with hospital leaders to move RRT nurses out of cardiology and into a new structure that became responsible for sepsis care quality. This effort was critical to ensure alignment in organizational priorities because otherwise, busy nurses would likely not consistently be able to make time to use (let alone offer feedback about) the sepsis AI tool. Generalizable insight: Invest time and energy in modernizing the organization to most effectively utilize emerging technologies like AI. In cases where an AI tool does not fit seamlessly within workflows or professional roles, the project team may need to be empowered to drive organizational change.
- *[Clinical Integration] Workflow burden*: During the rollout, iPads were prepared for both the RRT nurses and also patient workflow coordinator (PWC) nurses in the ED. However, after a few months post-clinical integration, the innovation team learned that the patient workflow coordinators were too busy with other duties to be using the sepsis AI tool; the model predictions were not as critical to PWC nurses’ immediate priorities. This process could have been streamlined by having regular check-ins with front-line workers, in addition to managers, to surface friction on the ground. Generalizable insight: Adapt the workflow to the needs of front-line workers and build flexibility into early pilots. Being able to respond to feedback also builds trust among front-line workers.

## Discussion

Three major insights derived from our study can inform future work. First, the algorithm journey map captures an extremely messy process that is far from ideal, and the effort required to surface the process is not scalable. In total, the algorithm journey map included 7 components for 4 stages and a separate table to capture all lifecycle management activities. Numerous interviews and co-development workshops were held with individuals who were involved in various stages of AI adoption and our team had to set bounds on the level of detail included in the journey map. Almost every step in the process could go further into detail to further explicate individual sub-steps. While the current study aimed to advance the understanding of traceability and transparency, we do not recommend that algorithm journey maps accompany every single instance of health AI adoption. This conclusion is different from Model Facts labels or data quality assurance documentation, which are recommended for all adopted health AI projects^21^.

Organizations and settings must then determine when concretization of AI adoption is most valuable. Unfortunately, while other organizations may learn from our experience, we do not expect that the algorithm journey map presented in this study maps well to any other setting or use case. By design, this traceability artifact is extremely enmeshed with our particular use case and setting. Other organizations may consider developing their own algorithm journey maps after completing an AI adoption process. We hope that as more groups publicly disseminate traceability artifacts like the algorithm journey map, organizations can learn from each other how to streamline the process and avoid common pitfalls.

The second major insight is the urgent need to standardize the health AI adoption process. The specific path visualized in the sepsis AI tool algorithm journey map is highly circuitous, confusing, and not meant to ever be repeated. Organizations, including ours, must actively streamline the process and define the most relevant and important decision points throughout the lifecycle stages. While developing the algorithm journey map, we did align the structure with 4 stages that can be adopted by other projects. Future work is needed to further align activities across healthcare delivery settings to define a standard process by which health AI tools can be adopted across settings. Hopefully, as different settings align their processes, the complexity of and effort required to develop algorithm journey maps will significantly decrease.

The third major insight is the immediate opportunity to leverage the concrete algorithm journey map to design traceability and transparency artifacts needed to facilitate the adoption of health AI tools. There’s limited understanding of the specific user, knowledge base, context-of-use, and decision made using information contained in the documentation artifact. With the algorithm journey map, researchers can now tailor documentation and transparency artifacts to specific decision points in a process. The user, use case, and implications become clear. While we do not recommend building out documentation for every single decision point, because many decision points are redundant or inefficient, we do expect that documentation efforts can target a small number of key decision points visualized throughout the process.

There are also three primary limitations of the current study. First, we focus on a single algorithm within a single setting. While many machine learning in healthcare studies seek to develop and validate frameworks across multiple models and settings, our objective was different. Rather than contribute additional documentation artifacts or well-organized processes for developing health AI tools, we address a core limitation of existing work. Our study breaks out of common pitfalls that limit visibility into complex sociotechnical processes, but in doing so we are myopically focused on minute details for a single use case. Our study does not address general questions such as the administrative roles, measures of AI tool viability, and form and frequency of communication required for AI tools to be successfully integrated. We address this limitation by identifying generalizable learnings that surfaced within the algorithm journey map, and we present key insights that are immediately informative to other groups.

Second, and relatedly, algorithm journey maps might emphasize a specific perspective, amplifying existing power structures rather than allowing less-powerful stakeholders to have their experiences properly reflected^22^. Further, a given algorithm journey map might not be able to capture all of the relevant context to scale across locations or time periods. Models of innovation are dependent on political, cultural, and institutional factors, requiring a high degree of contextual specificity in each case study^23,24^.

The third limitation is a lack of standardization for the amount of detail to include in a journey map. We scoped the journey map to not include any activities prior to problem selection for the sepsis AI tool, even though previous technologies were used to detect sepsis. We also did not include any new sepsis models developed as separate projects during lifecycle management. Within the steps depicted in the algorithm journey map, we included details relevant to the reader, but some may find the level of detail excessive or insufficient. We hope that as more organizations disseminate traceability artifacts, standards emerge for how to best visualize algorithm journey maps.

We hope that in future work, additional organizations can build upon this approach of making these processes more tangible. As more case studies are fleshed out, it will be easier to normatively discuss the best way to operationalize other ethical principles. For instance, the AI sepsis tool adoption captured by this journey map did not include patients at any step, and – unlike for the cases of nurses—that idea did not even come up during the interviews as an opportunity to reflect on where they should have been included. Many projects in this field also have struggled with this question around patient inclusion; as more projects are more tangible, the field will be able to learn from what methods of patient engagement (and other instantiations of ethical computing principles) are more/less meaningful.

## Methods

### Data collection

In this study, we document the effort required to build and integrate an ML-based sepsis detection algorithm in a large health system. The purpose of this study was to identify the stakeholders and decisions that were made throughout the entire effort in order to develop documentation artifacts that would be helpful for people in those roles for future projects. In order to understand this effort, our algorithm journey map is composed of workflow diagrams to track the pre-deployment efforts because of their mostly linear nature and then a table of the post-deployment efforts due to their concurrent and asynchronous nature.

We interviewed every stakeholder on the innovation team (Duke Institute for Health Innovation) who was involved in the project. Six participants with the roles of a clinical data scientist, an innovation program manager, a data engineer, two solutions architects, and an innovation program director were recruited. In total, we conducted 20 unstructured interviews with them to gather information about each stage featured in the algorithm journey map. Although there was no interview guide, each participant was asked to describe:

- Which stakeholders were involved in that part of the project?
- What work was done (and by whom) for the parts of the project they worked on?
- What decisions were made?
- What data/information was used to make those decisions?
- What decision points weren’t considered but should have been?

During early interviews, we either took detailed notes or interactively built visual schematics using Microsoft Visio, which is a diagramming and vector graphics application. We then met with the sepsis project leaders multiple times to clarify questions and refine the algorithm journey map.

### Process mapping

A process map is an artifact that documents a workflow, allowing decision-makers to identify the steps, stakeholders, and decisions made during a given process or activity. There can be many variants of the concept, such as basic flowcharts, customer journey map^25^, value stream map^26^, and more. A recent systematic review that evaluated 105 process mapping manuscripts found that process maps help to support understanding of complex healthcare systems and can guide improvement efforts within their local context^27^. de Ven articulates how a process map’s narrative presentation is well-suited to show the complexities of a workflow, which can be nonlinear and have unexpected twists and turns^28^. Adopting this awareness is especially important to implementing technologies in organizational settings, which often require changes to workers’ roles, relationships, and authority structures^29,30^.

To construct the pre-deployment section of the algorithm journey map, we use a visualization technique from the process map literature, namely swimlane diagrams^18^. Damelio describes a swimlane diagram as “a set and series of interrelated work activities and resources that follow a distinct path as work inputs (resources) get transformed into outputs (items) that customer’s value” and such diagrams are used to delineate the various stakeholders and their interactions^26^.

This study was a quality improvement project with minimal risk to the participants. IRB review and approval was not required according to Duke University policy, because the project is not research that is subject to federal human subjects protection regulations^31^. The activities carried out as part of this project can improve the process for algorithm implementation locally, the project evaluates the current practices of clinicians and staff directly involved in the project, and future patients can benefit from improvements in the process. The project followed the ethical principles of research and the privacy, confidentiality, and autonomy of all participants were respected. All participants were informed about the purpose of the project before contributing the information needed, and no harm was anticipated or reported due to participation. All staff who were interviewed for the project provided consent prior to interviews.

## Data Availability

Much of the materials or information used to inform this study have been previously published or made available on the Duke Institute for Health Innovation website. Requests for material used in this study that is not currently publicly available can be sent to the corresponding author.
